# *In Vitro* Activities of Aztreonam-Avibactam, Eravacycline, Cefoselis, and Other Comparators against Clinical *Enterobacterales* Isolates: a Multicenter Study in China, 2019

**DOI:** 10.1128/spectrum.04873-22

**Published:** 2023-05-15

**Authors:** Jiawei Chen, Yong Liu, Wei Jia, Xuesong Xu, Guizhen Sun, Tong Wang, Jin Li, Ge Zhang, Ran Jing, Hongli Sun, Yingchun Xu, Yali Liu

**Affiliations:** a Department of Laboratory Medicine, State Key Laboratory of Complex Severe and Rare Diseases, Peking Union Medical College Hospital, Chinese Academy of Medical Sciences and Peking Union Medical College, Beijing, China; b Graduate School, Peking Union Medical College, Chinese Academy of Medical Sciences, Beijing, China; c Department of Clinical Laboratory, Shengjing Hospital of China Medical University, Shenyang, China; d Medical Experimental Center, General Hospital of Ningxia Medical University, Yinchuan, China; e China-Japan Union Hospital, Jilin University, Changchun, China; f Department of Clinical Laboratory, Beijing Youan Hospital, Capital Medical University, Beijing, China; JMI Laboratories

**Keywords:** aztreonam-avibactam, eravacycline, cefoselis, multidrug-resistant *Enterobacterales*, carbapenem-resistant *Enterobacterales*, antimicrobial susceptibility testing

## Abstract

Aztreonam-avibactam, eravacycline, and cefoselis are three novel antimicrobial agents for the treatment of serious infections caused by Gram-negative bacteria. We evaluated the *in vitro* activities of the above-mentioned three antimicrobial agents against clinical *Enterobacterales* isolates. A total of 1,202 *Enterobacterales* isolates, including 10 genera or species, were collected from 26 hospitals that cover seven regions of China. The susceptibilities of the 30 antimicrobial agents were interpreted based on the combination of U.S. Food and Drug Administration and Clinical and Laboratory Standards Institute guidelines. The results indicated that all *Enterobacterales* isolates showed high susceptibility to aztreonam-avibactam (98.25%), eravacycline (85.69%), and cefoselis (62.73%). The first two antimicrobial agents also demonstrated potent activities against multidrug-resistant and carbapenem-resistant *Enterobacterales* independent of antimicrobial resistance mechanisms. The rates of susceptibility to aztreonam-avibactam, eravacycline, and cefoselis were lowest in *Morganella* spp. (84.42%), Proteus spp. (33.65%), and Escherichia coli (40.14%), respectively. In general, the lower rates of susceptibility to eravacycline and cefoselis were in the older inpatient group. The strains isolated from urinary tract exhibited the lowest rate of susceptibility (78.97%) to eravacycline, and the lowest rate of susceptibility (45.83%) to cefoselis was observed in nervous system specimens. The strains isolated from intensive care unit (ICU) wards showed significantly reduced susceptibility to cefoselis compared with those isolated from non-ICU wards. The MIC values of aztreonam-avibactam and ceftazidime-avibactam have poor consistency (weighted kappa = 0.243), as did eravacycline and tigecycline (weighted kappa = 0.478). Cefoselis and cefepime showed highly similar activities against *Enterobacterales* (weighted kappa = 0.801). Our results support the clinical development of aztreonam-avibactam, eravacycline, and cefoselis to treat infections caused by *Enterobacterales*.

**IMPORTANCE** Infections caused by multidrug-resistant (MDR) *Enterobacterales*, especially carbapenem-resistant *Enterobacterales* (CRE), have been a challenging clinical problem due to the limited therapeutic options. Therefore, the need to develop novel antimicrobial agents and evaluate their activities against *Enterobacterales in vitro* is urgent. Our results show that the novel antimicrobial agents aztreonam-avibactam and eravacycline retain activities against MDR and CRE isolates, including carbapenemase producers and non-carbapenemase producers. Further analysis combined with clinical information on the strains tested revealed that no significant differences were observed in susceptibility rates of strains with different demographic parameters to aztreonam-avibactam. Age, specimen source, and department were associated with the susceptibility of strains to eravacycline and cefoselis (*P* ≤ 0.01). Compared with ceftazidime-avibactam, aztreonam-avibactam has its advantages and limitations against *Enterobacterales*. The potent activity of eravacycline against *Enterobacterales* was higher than that of tigecycline. Cefoselis and cefepime showed a highly consistent activity against *Enterobacterales*.

## INTRODUCTION

Antimicrobial resistance is becoming an increasing threat endangering global public health. Multidrug-resistant (MDR) pathogens can increase the disease burden and cause severe infections associated with high morbidity and mortality (1). *Enterobacterales* are among the most prominent pathogens associated with antimicrobial resistance, as the pathogens are common causes of community-associated and health care-associated infections. Carbapenems possess the broadest antimicrobial spectrum of activity and are the favored last-resort drugs for treatment of MDR *Enterobacterales* infections. Worryingly, the emergence and worldwide dissemination of carbapenem-resistant *Enterobacterales* (CRE) pose one of the most challenging threats to human health (2). The main mechanisms of carbapenems resistance in CRE include (i) production of carbapenemases and (ii) production of extended-spectrum β-lactamases (ESBLs) or AmpC β-lactamases combined with the loss of either of the major outer membrane proteins (OMPs) porin and upregulated efflux pumps (3). In light of this, developing novel antimicrobial agents to combat the threat of these emerging resistant bacteria is urgent (4).

Aztreonam belongs to the member of the monobactam class and was approved by the U.S. Food and Drug Administration (FDA) in 1986 (5). Although aztreonam is stable to hydrolysis by metallo-β-lactamases (MBLs) compared with other β-lactams, it can also be hydrolyzed by most clinically relevant serine lactamases, such as ESBLs, AmpC β-lactamases, and Klebsiella pneumoniae carbapenemases (KPCs) (6). Its combination with avibactam, a novel non-β-lactam β-lactamase inhibitor which can effectively inhibit serine lactamases, has potential utility against MDR bacterial pathogens and CRE (7, 8). Eravacycline is a novel broad-spectrum synthetic tetracycline antibiotic comprising a tetracycline core with two novel modifications, a fluorine atom at the C-7 position and apyrrolidinoacetamido group at the C-9 position (9). Eravacycline inhibits bacterial protein synthesis through binding to the 30S ribosomal subunit and retains antibacterial activities against isolates with tetracycline ribosomal protection proteins or most tetracycline efflux proteins, the same as tigecycline (10). Cefoselis is a fourth-generation cephalosporin with a 1-hydroxyethyl-5-aminopyrazole moiety at position 3 of the cephem ring (11). Due to its structural properties, cefoselis has a higher antibacterial activity than the third-generation cephalosporins against *Enterobacterales* (12).

Awareness of local antimicrobial susceptibility data plays a guiding role in selecting drugs to treat infections. Currently, only a few reports have been published in China on the *in vitro* antimicrobial activities of aztreonam-avibactam, eravacycline, and cefoselis against *Enterobacterales* isolates. This multicenter study in China was conducted to evaluate the *in vitro* activities of these three antimicrobial agents against a large collection of clinical *Enterobacterales* isolates recovered from hospitalized patients and to understand the demographic parameters which affect the susceptibilities of *Enterobacterales* isolates to these agents.

## RESULTS

**FIG 1:**
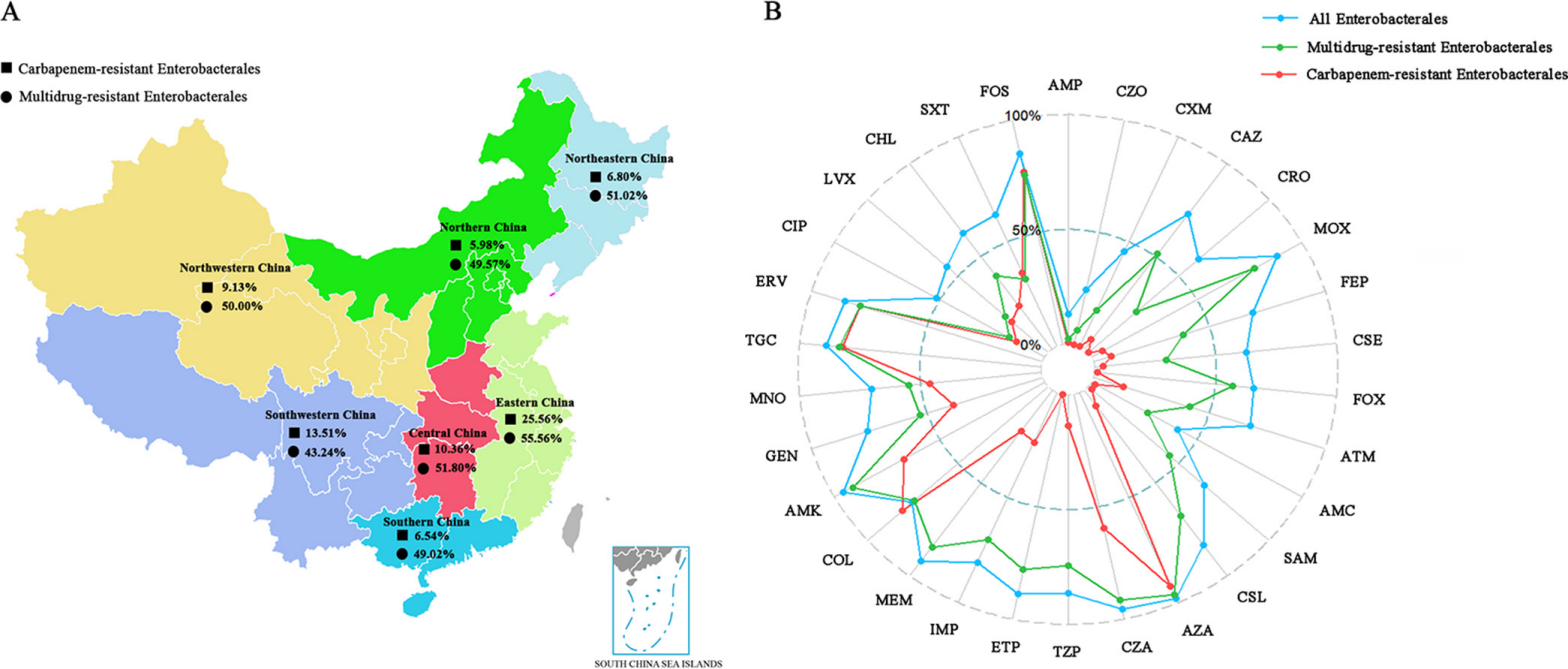
(A) Frequency of MDR and CRE isolates in various regions in China. (B) Rates of susceptibility of all *Enterobacterales*, MDR, and CRE isolates to 30 antimicrobial agents.

**FIG 2:**
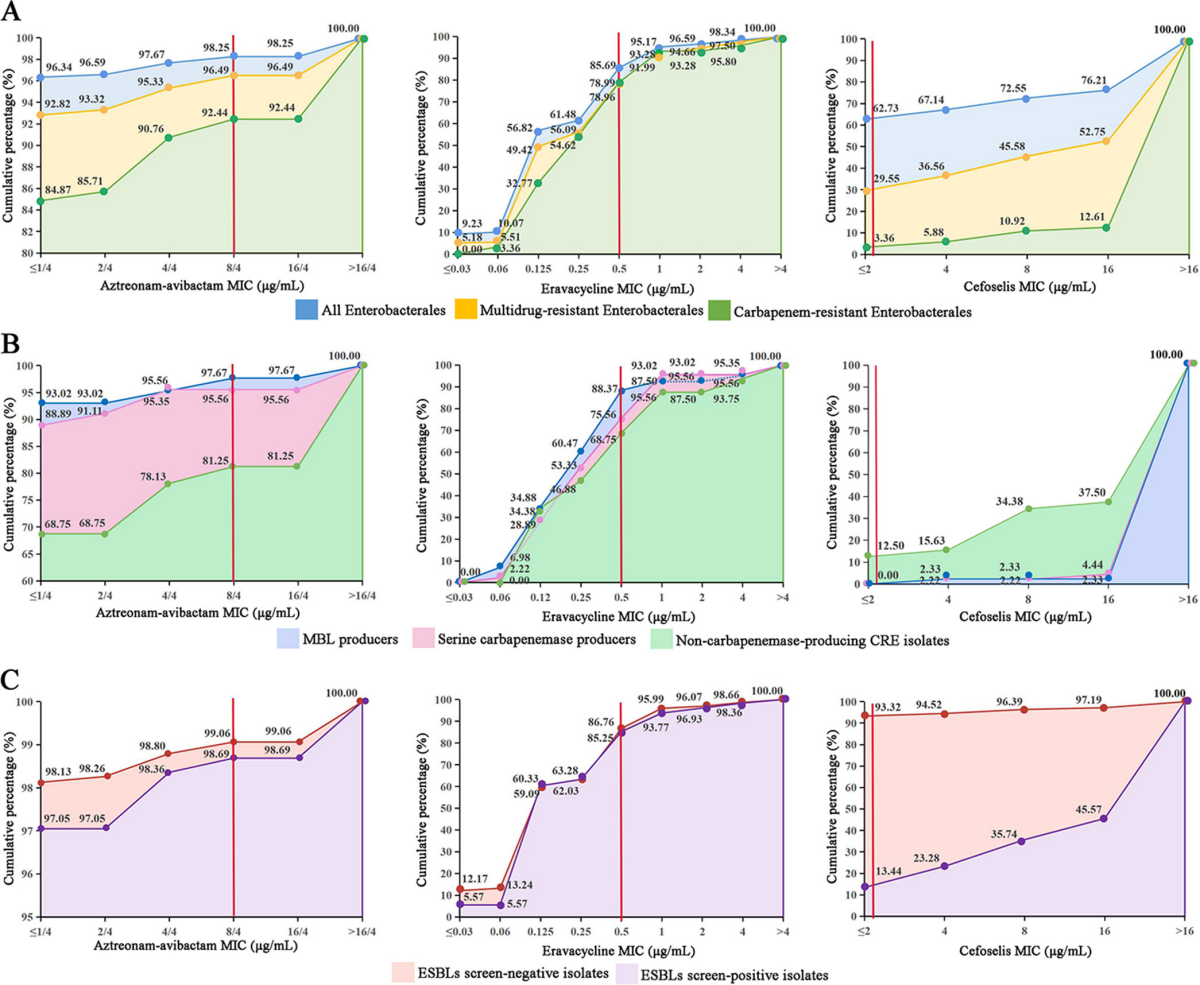
MIC cumulative frequency distribution of aztreonam-avibactam, eravacycline, and cefoselis against three organism groups: all *Enterobacterales*, MDR, and CRE isolates (A), serine carbapenemase producers, MBL producers, and non-carbapenemase-producing CRE isolates (B), and ESBL screen-positive isolates and screen-negative isolates (C). The red line represents the susceptibility breakpoints of the corresponding antimicrobial agents.

Among the 1,202 *Enterobacterales* isolates in this study, Escherichia (including only Escherichia coli) was the most abundant genus (*n* = 284), followed by Klebsiella (*n* = 243), Salmonella (*n* = 169), Enterobacter (*n* = 140), Proteus (*n *= 104), *Serratia* (*n = *97), *Citrobacter* (*n = *82), *Morganella* (*n *= 77), *Providencia* (*n *= 5), and *Edwardsiella* (*n *= 1). These isolates were collected from different specimens, including blood (*n *= 241) and respiratory tract (*n *= 191), abdominal cavity (*n *= 94), skin and soft tissue (*n *= 108), urinary tract (*n *= 214), gastrointestinal tract (*n *= 187), genital tract (*n *= 119), and nervous system (*n *= 48). The frequency of MDR isolates in various regions in China ranged from 43.24% to 55.56% (49.83% overall). The frequency of CRE isolates was highest in eastern China (25.56%), followed by southwestern China (13.51%), central China (10.36%), northwestern China (9.13%), northeastern China (6.80%), southern China (6.54%), and northern China (5.98%) (Fig. 1A). Among 119 CRE isolates, a total of 88 carbapenemases produced by 87 isolates were detected (1 K. pneumoniae isolate produced two carbapenemases, KPC-2 and NDM-1), including 43 KPCs (43 KPC-2), 40 NDMs (22 NDM-1, 16 NDM-5, 1 NDM-4, and 1 NDM-9), 3 IMPs (1 IMP-4, 1 IMP-8, and 1 IMP-26), and 2 OXAs (1 OXA-181 and 1 OXA-232). Through ESBL screening and confirmation tests, we detected 307 ESBL screen-positive isolates and 748 screen-negative isolates. We assessed the *in vitro* activities of 30 antimicrobial agents against all *Enterobacterales* isolates tested, including MDR and CRE isolates, and the results are listed in Table 1. Overall, all *Enterobacterales* isolates retained high rates of susceptibility to aztreonam-avibactam (98.25%), amikacin (95.76%), ceftazidime-avibactam (95.76%), and meropenem (92.26%). Only 13.14% and 24.46% of isolates in our study were susceptible to ampicillin and cefazolin, respectively. Other antimicrobial agents showed susceptibility rates ranging from 41.01% to 89.02%. The most active agents tested against MDR and CRE isolates were aztreonam-avibactam (susceptibility rates of 96.49% and 92.44%, respectively), amikacin (susceptibility rates of 91.49% and 67.23%, respectively), ceftazidime-avibactam (susceptibility rates of 91.49% and 59.66%, respectively), tigecycline (susceptibility rates of 83.81% and 82.35%, respectively), colistin (susceptibility rates of 74.12% and 80.67%, respectively), eravacycline (susceptibility rates of 78.96% and 78.99%, respectively), and fosfomycin (susceptibility rates of 75.79% and 77.00%, respectively) (Fig. 1B and Table 1). To further investigate the *in vitro* activities of the three novel antimicrobial agents, aztreonam-avibactam, eravacycline, and cefoselis, against MDR and CRE isolates, we analyzed the MIC distributions and the cumulative percentage of these three novel antimicrobial agents against three organism groups, (i) all *Enterobacterales*, MDR, and CRE isolates, (ii) serine carbapenemase producers, MBL producers, and non-carbapenemase-producing CRE (the K. pneumoniae isolate coproducing KPC and NDM belongs to both the serine carbapenemase producer and MBL producer groups), and (iii) ESBL screen-positive isolates and screen-negative isolates (Fig. 2). Aztreonam-avibactam inhibited 97.67% of isolates at 4/4 μg/mL, including 95.33% of MDR isolates and 90.76% of CRE isolates. Moreover, 96.34% of isolates, including 92.82% of MDR isolates and 84.87% of CRE isolates, had aztreonam-avibactam MIC values of ≤1/4 μg/mL. Eravacycline also was highly effective against all isolates, with an 85.69% susceptibility rate, and the MIC_50_ and MIC_90_ values were 0.125 and 1 μg/mL, respectively. Similarly, eravacycline was highly effective against MDR and CRE isolates, both with MIC_50/90_ values of 0.25/1 μg/mL. Cefoselis MIC values ranged from ≤2 to 16 μg/mL and showed a comparatively low susceptibility rate (62.73%) for all isolates. Only 45.58% of MDR isolates and 10.92% of CRE isolates were inhibited by cefoselis at a MIC value of 8 μg/mL (Fig. 2). Among CRE isolates, both aztreonam-avibactam and eravacycline had high activities against carbapenemase (serine carbapenemases and MBLs) and non-carbapenemase producers, with all susceptibility rates exceeding 68%. Further observation revealed that eravacycline exhibited similar MIC distributions against both carbapenemase producers and non-carbapenemase producers, while the MIC distributions of aztreonam-avibactam and cefoselis against these two types of CRE differed. Non-carbapenemase producers showed a comparatively lower rate of susceptibility to aztreonam-avibactam (81.25%) and a relatively higher rate of susceptibility to cefoselis (12.50%) than did carbapenemase producers (Fig. 2). Similar to the case with ESBL screen-negative isolates, both aztreonam-avibactam and eravacycline were also highly active against ESBL screen-positive isolates, with MIC_90_ values of ≤1/4 μg/mL and 1 μg/mL, respectively. Inevitably, ESBL screen-negative isolates showed very high rates of susceptibility to cefoselis (93.92%) (Fig. 2).

**TABLE 1:** Activities of 30 antimicrobial agents against 1,202 tested isolates, including MDR and CRE isolates

Antimicrobial agent	All isolates (*n *= 1,202)			MDR isolates (*n *= 599)			CRE isolates (*n *= 119)		
	IC_50_ (μg/mL)	IC_90_ (μg/mL)	S^*a*^ (%)	IC_50_ (μg/mL)	IC_90_ (μg/mL)	S (%)	IC_50_ (μg/mL)	IC_90_ (μg/mL)	S (%)
Ampicillin	>16	>16	13.14	>16	>16	2.50	>16	>16	0.84
Cefazolin	>16	>16	24.46	>16	>16	6.34	16	>16	0.00
Cefuroxime	16	>64	45.26	>64	>64	17.20	>64	>64	0.00
Ceftazidime	≤1	>64	72.96	4	>64	51.42	>64	>64	5.04
Ceftriaxone	≤0.25	>64	61.06	>64	>64	26.71	>64	>64	0.00
Moxalactam	≤2	32	88.10	≤2	>32	77.63	>32	>32	5.04
Cefepime	≤0.5	>16	68.97	8	>16	38.56	>16	>16	7.56
Cefoselis	≤2	>16	62.73	16	>16	29.55	>16	>16	3.36
Cefoxitin	8	>16	65.64	8	>16	56.93	>16	>16	0.84
Aztreonam	≤1	>16	67.89	16	>16	41.57	>16	>16	12.61
Amoxicillin-clavulanate	16/8	>16/8	41.01	>16/8	>16/8	26.71	>16/8	>16/8	1.68
Ampicillin-sulbactam	16/8	>16/8	64.06	>16/8	>16/8	44.91	>16/8	>16/8	1.68
Cefoperazone-sulbactam	2/1	64/32	83.36	8/4	>64/32	67.78	>64/32	>64/32	8.40
Aztreonam-avibactam	≤1/4	≤1/4	98.25	≤1/4	≤1/4	96.49	≤1/4	4/4	92.44
Ceftazidime-avibactam	≤0.5/4	1/4	95.76	≤0.5/4	4/4	91.49	4/4	>16/4	59.66
Piperacillin-tazobactam	4/4	64/4	86.44	4/4	>128/4	74.46	>128/4	>128/4	13.45
Ertapenem	≤0.125	1	88.85	≤0.125	>8	77.96	>8	>8	0.00
Imipenem	≤0.25	4	80.95	≤0.25	32	70.12	32	>32	23.53
Meropenem	0.064	1	92.26	0.125	32	84.47	32	>32	21.85
Colistin^*b*^	2	>16	75.29	2	32	74.12	1	>16	80.67
Amikacin	2	4	95.76	4	16	91.49	4	>32	67.23
Gentamicin	1	>8	75.79	2	>8	53.09	>8	>8	38.66
Minocycline	4	>8	70.38	4	>8	54.92	8	>8	46.22
Tigecycline	0.5	4	89.02	0.5	4	83.81	0.5	4	82.35
Eravacycline	0.125	1	85.69	0.25	1	78.96	0.25	1	78.99
Ciprofloxacin	0.25	>2	51.66	>2	>2	17.03	>2	>2	13.45
Levofloxacin	0.5	>4	56.16	4	>4	23.71	>4	>4	20.17
Chloramphenicol	8	>16	62.65	>16	>16	39.57	>16	>16	23.53
Trimethoprim-sulfamethoxazole	≤0.5/9.5	>4/76	62.90	>4/76	>4/76	32.05	>4/76	>4/76	35.29
Fosfomycin	≤2	128	85.36	4	>128	75.79	64	>128	77.00

a S, susceptibility.

b The susceptibility rate of colistin was replaced with the nonresistance rate.

**FIG 3:**
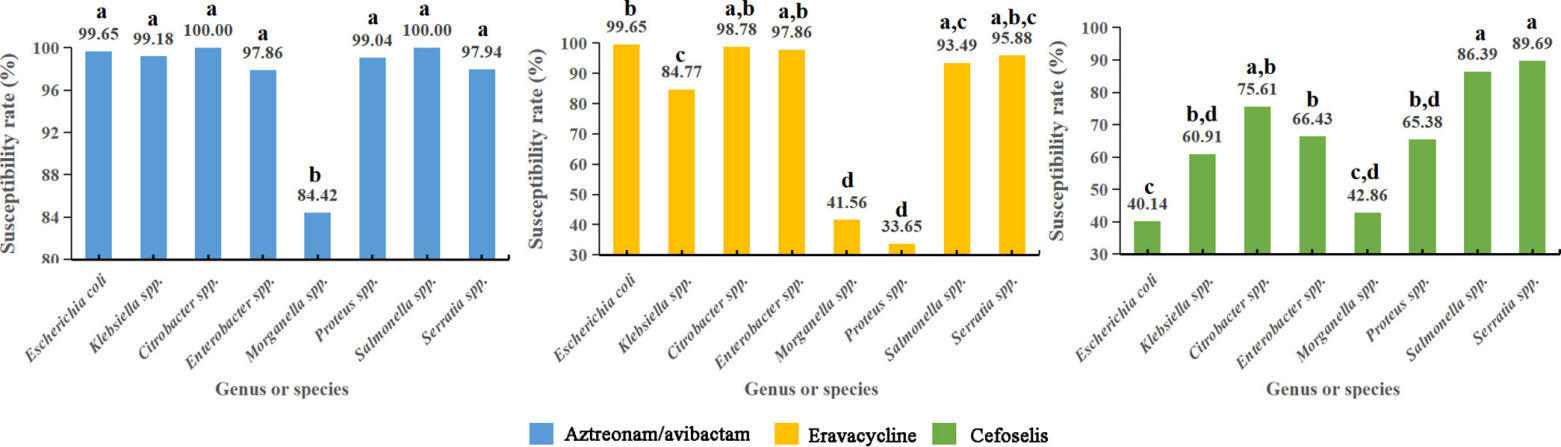
Susceptibility rates of different species, including Escherichia coli, Klebsiella spp., Salmonella spp., Enterobacter spp., Proteus spp., *Serratia* spp., *Citrobacter* spp. and *Morganella* spp., to aztreonam-avibactam, eravacycline, and cefoselis. The same letters for a given antimicrobial agent indicate that no significant differences were observed in susceptibility rate at a *P* value of ≤0.05.

Figure 3 shows the rates of susceptibility of different species and genera of *Enterobacterales* to the three novel antimicrobial agents. The number of *Providencia* spp. and *Edwardsiella* spp. was too small to be included in the analysis. Overall, aztreonam-avibactam was active against all individual species and genera. All species and genera displayed very high rates of susceptibility to aztreonam-avibactam, ranging from 97.86% to 100.00%, except for *Morganella* spp., which exhibited a relatively low susceptibility rate (84.42%) (*P* ≤ 0.01). Similarly, aztreonam-avibactam MIC_50_ and MIC_90_ values against all species and genera except for *Morganella* spp. were ≤1/4 μg/mL (see Table S1 in the supplemental material). For eravacycline, *Morganella* spp. and Proteus spp. exhibited significantly lower susceptibility rates than did other genera or species (*P* ≤ 0.01). A total of 58.44% of *Morganella* spp. and 66.35% of Proteus spp. exhibited nonsusceptible MIC values for eravacycline (>0.5 μg/mL). The highest MIC_50_ value (1 μg/mL) and MIC_90_ (8 μg/mL) of eravacycline were both observed in Proteus spp. Because eravacycline and tigecycline have similar structures, Proteus and *Morganella*, which are intrinsically resistant to tigecycline, could be also less susceptible to eravacycline than other genera of *Enterobacterales* (13). Cefoselis, as an agent belonging to the fourth-generation cephalosporins, was slightly active against E. coli, with a susceptibility rate of 40.14%. Likewise, E. coli also exhibited the lowest rates of susceptibility to ceftriaxone and cefepime (34.51% and 42.25%) in this study. E. coli is a very common species producing ESBLs, especially some clonal lineages, such as E. coli sequence type (ST) 131, which often produces CTX-M group ESBLs (14).

Clinical information for the 1,202 hospitalized patients was included in this study to assess the effects of different demographic parameters (gender, region, age, specimen source, and department) associated with the resistance of *Enterobacterales* isolates to the three novel antimicrobial agents, aztreonam-avibactam, eravacycline, and cefoselis. No significant differences in rates of susceptibility to aztreonam-avibactam were observed among different demographic parameters (Table 2). For eravacycline and cefoselis, gender and region had no significant effect on the susceptibility rates of the corresponding isolates, but the isolates showed significantly different susceptibilities to eravacycline and cefoselis in the different age and specimen source groups. With the increasing ages of the inpatients, *Enterobacterales* isolates had reduced susceptibility to eravacycline and cefoselis. The one exception to this pattern was the isolates from patients age group ≤14 years, which showed the lowest rate of susceptibility to cefoselis. Among different specimen sources, the isolates from the urinary tract showed the lowest rate of susceptibility to eravacycline (78.97%), and the lowest rate of susceptibility to cefoselis (45.83%) was observed in the nervous system. Compared with non-ICU wards, the isolates collected from ICU wards had reduced susceptibility only to cefoselis among the three novel antimicrobial agents.

**TABLE 2:** Susceptibilities of tested *Enterobacterales* isolates with different demographic parameters to aztreonam-avibactam, eravacycline, and cefoselis

Demographic parameter	Aztreonam-avibactam		Eravacycline		Cefoselis	
	S (%)	Difference^*a*^	S (%)	Difference	S (%)	Difference
Gender						
Female	98.31	a	86.12	a	63.98	a
Male	98.21	a	85.35	a	61.73	a
Region						
Northeastern China	97.96	a	87.07	a	57.14	a
Northwestern China	98.56	a	85.10	a	62.02	a
Southwestern China	99.32	a	89.19	a	63.51	a
Northern China	97.86	a	86.32	a	67.95	a
Southern China	98.04	a	80.39	a	66.01	a
Central China	96.85	a	82.88	a	63.96	a
Eastern China	98.89	a	93.33	a	50.00	a
Age (yrs)						
≤14	98.51	a	94.06	a	55.94	a
15–35	98.67	a	86.67	a, b	77.33	b
36–60	97.18	a	84.27	b	63.38	a
≥61	99.06	a	82.78	b	60.14	a
Specimen source						
Respiratory tract	99.48	a	88.48	a, b	63.35	a, b, c
Abdominal cavity	98.94	a	86.17	a, b	52.13	a, b
Blood	98.34	a	87.55	a, b	71.78	c
Skin and soft tissue	98.15	a	82.41	a, b	65.74	a, b, c
Urinary tract	96.26	a	78.97	b	51.87	b
Gastrointestinal tract	98.40	a	91.44	a	66.31	a, b, c
Genital tract	99.16	a	78.99	a, b	69.75	a, c
Nervous system	97.92	a	95.83	a, b	45.83	a, b
Department						
ICU	99.25	a	84.21	a	45.86	a
Non-ICU	98.13	a	85.87	a	64.83	b

a The same letters for the same parameter indicate that no significant differences were observed in susceptibility rate at a *P* value of ≤0.05.

**FIG 4:**
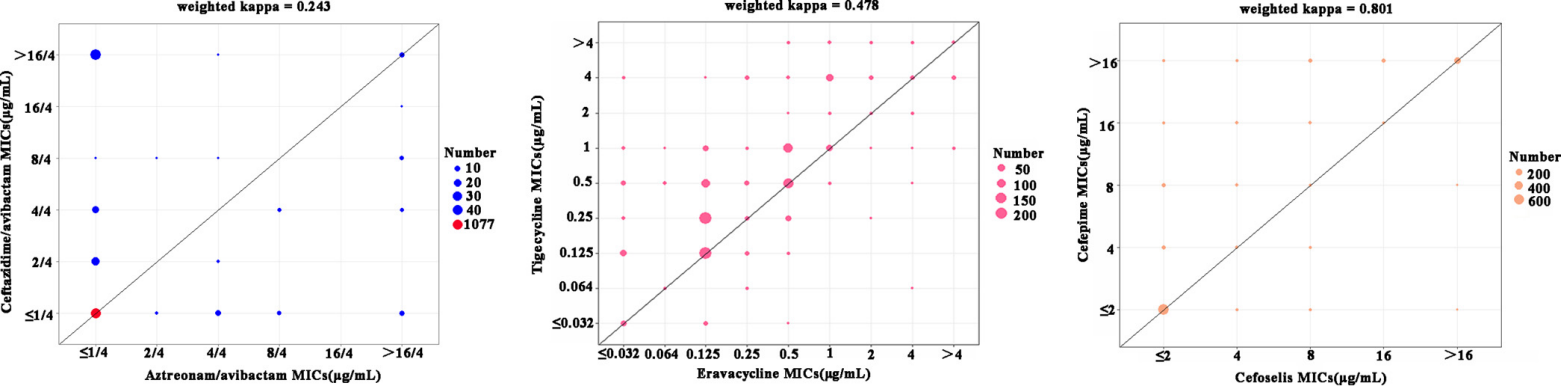
MIC values of aztreonam-avibactam, eravacycline, and cefoselis versus three comparators, ceftazidime-avibactam, tigecycline, and cefepime, against all *Enterobacterales* isolates. The central line indicates that the strains shared identical MIC values for two comparative antimicrobial agents.

A scatterplot with the distribution of MIC values was used to assess the potency of the three novel antimicrobial agents (aztreonam-avibactam, eravacycline, and cefoselis) versus three comparators (ceftazidime-avibactam, tigecycline, and cefepime) against 1,202 *Enterobacterales* isolates (Fig. 4). Because of the high antibacterial activities of aztreonam-avibactam and ceftazidime-avibactam, most of the MIC values of the two antimicrobial agents were ≤1/4 μg/mL. It also should be noted that a few (*n *= 43) ceftazidime-avibactam MIC values were >16/4 μg/mL when the corresponding aztreonam-avibactam MIC values were ≤1/4 μg/mL. In contrast, there are 35 cases in which MIC values of aztreonam-avibactam were higher than those of ceftazidime-avibactam. The analysis of linear weighted kappa further indicated that the MIC values of aztreonam-avibactam and ceftazidime-avibactam had poor consistency against isolates tested (weighted kappa = 0.243). The comparison of antibacterial activities of eravacycline with tigecycline also exhibited diversity (weighted kappa = 0.478). Most of the tigecycline MIC values were higher than the corresponding MIC values of eravacycline (2- to 4-fold in many cases). For cefoselis and cefepime, although a few MIC values of cefoselis were higher than that of cefepime, 80.78% of strains shared identical cefoselis and cefepime MIC values. A linear weighted kappa value of 0.801 showed good agreement between cefoselis and cefepime MIC values.

## DISCUSSION

In recent years, the prevalence of CRE has significantly increased in the world (15). In this study, CRE rates varied by geography and the highest CRE rate was 25.56% in eastern China, the most economically developed region of China. Yet therapeutic options for CRE infections have been limited. A few agents, such as colistin, tigecycline and novel β-lactam-β-lactamase inhibitor combinations, ceftazidime-avibactam, meropenem-vaborbactam, and imipenem-relebactam, may remain active against these organisms. However, ceftazidime-avibactam, meropenem-vaborbactam, and imipenem-relebactam are effective against KPC-producing CRE isolates, while they have limited effectiveness against OXA- and MBL-producing CRE isolates (16). In this study, aztreonam-avibactam and eravacycline showed considerable effectiveness against MDR and CRE isolates. The aztreonam-avibactam MIC_90_ values among CRE and MDR isolates were only 4/4 μg/mL and ≤1/4 μg/mL, respectively, and eravacycline MIC_90_ values were both 1 μg/mL. Further, the aztreonam-avibactam and eravacycline MICs reflected potent activities against CRE isolates independent of their carbapenem resistance mechanisms, including the production of KPCs, OXAs, and MBLs or other resistance mechanisms. Our study results were similar to those in previous publications by other investigators. Sader et al. evaluated the *in vitro* activity of aztreonam-avibactam against 8,787 *Enterobacterales* isolates, including 396 CRE isolates and 1,706 MDR isolates, and reported that aztreonam-avibactam MIC_90_ values against CRE and MDR isolates were both 0.5/4 μg/mL. In addition, CRE isolates stratified by carbapenemases type all had aztreonam-avibactam MIC_50_ values of 0.12 to 0.25/4 μg/mL (17). A Chinese study published by Zhao et al. revealed that eravacycline MIC_90_ values for carbapenem-resistant E. coli and Enterobacter cloacae were 1 μg/mL and 0.5 μg/mL, respectively. The production of various carbapenemases (KPC-2, NDM-1, and OXA-23) did not affect the activities of eravacycline against CRE isolates (18). Therefore, aztreonam-avibactam and eravacycline, developed for the treatment of complicated abdominal infections, demonstrate high activities against most clinically important antibiotic-resistant pathogens, including MDR and CRE pathogens (19–21). In addition, an unexpected observation was that non-carbapenemase-producing CRE strains (81.25%) were slightly less susceptible to aztreonam-avibactam than carbapenemase producers (serine carbapenemase producers, 95.56%, and MBL producers, 97.67%) (Fig. 2). An evaluation study for the *in vitro* efficacy of a broad spectrum of antibiotics against CRE from the Arabian Peninsula also revealed a lower rate of susceptibility of non-carbapenemase producers to aztreonam-avibactam than that of carbapenemase producers (85.4% versus 96.9%) (22). The relatively low susceptibility of non-carbapenemase-producing CRE isolates to aztreonam-avibactam may be due to the production of CMY-type AmpC β-lactamases, as Ma et al. found that the production of CMY-42 is correlated with aztreonam-avibactam resistance (23).

Aztreonam-avibactam, as a novel β-lactamase–inhibitor combination, is currently under clinical development (ClinicalTrials registration no. NCT01689207). Since aztreonam-avibactam is not used in the clinic, our results showed that the corresponding strains in different demographic parameter groups showed no significant differences in rates of susceptibility to aztreonam-avibactam. Eravacycline was approved by FDA and the European Medicines Agency (EMA) in 2018, but the approval is still pending in China. The strains with only two demographic parameters, the age of patients and specimen source, have significant differences in susceptibilities to eravacycline. Our results showed that the rate of susceptibility of isolates from urinary specimens to eravacycline was the lowest, followed by those from genital tract and skin and soft tissue specimens. This was due to relatively higher isolation rates of Proteus spp. and *Morganella* spp., which had low rates of susceptibility to eravacycline in urinary (27.10%), genital tract (21.85%), and skin and soft tissue specimens (20.37%) compared to less than 11% in other specimens (data not shown). Eravacycline was approved for the treatment of complicated intra-abdominal infections (cIAI) (24, 25). In this study, eravacycline exhibited a high activity against strains collected from the abdominal cavity, with a susceptibility rate of 86.17%. Cefoselis, as a novel fourth-generation cephalosporin, was widely applicable for respiratory, urogenital, and abdominal infections (11, 26). The susceptibility rates of corresponding strains from the respiratory tract, urinary tract, genital tract, and abdominal cavity to cefoselis were 63.35%, 51.87%, 69.75%, and 52.13%, respectively. A significant difference was observed in the activity of cefoselis against *Enterobacterales* isolates between ICU (susceptibility rate of 45.86%) and non-ICU wards (susceptibility rate of 64.83%). This may be related to the fact that rates of isolation of MDR (63.91%) and CRE (19.55%) strains from ICU wards were both significantly higher than those from non-ICU wards, at 48.08% and 8.70%, respectively (Table S2). A meta-analysis revealed that length of stay in the ICU wards is one risk factor related to infection and/or colonization with ESBL-producing bacteria (27). A study by Hu et al. also showed that the rate of isolation of carbapenem-resistant K. pneumoniae from ICU wards was significantly higher than that from non-ICU wards (28).

Ceftazidime-avibactam, as the only avibactam-based combination currently available for clinical use, represented an important advance in the treatment for infections caused by CRE (29). Aztreonam-avibactam has a broader spectrum of activity than ceftazidime-avibactam, with additional coverage for MBLs. In this study, ceftazidime-avibactam MIC values for 43 isolates were >16/4 μg/mL, while corresponding aztreonam-avibactam MIC values were ≤1/4 μg/mL. As expected, 95.35% (41/43) of these isolates produced MBLs. However, aztreonam-avibactam may not replace ceftazidime-avibactam. The production of KPC-21 in combination with four extra amino acids in penicillin-binding protein 3 (PBP3) is able to confer on strains resistance to aztreonam-avibactam, but they remain susceptible to ceftazidime-avibactam (30). Our results also showed that partial MIC values (*n *= 35) of aztreonam-avibactam were higher than the corresponding MIC values of ceftazidime-avibactam. The consistency test also showed possible different resistance mechanisms of the strains to the two antimicrobial agents. Therefore, the avibactam-based agents both have their limitations, and combination with ceftazidime-avibactam and aztreonam may be a good treatment strategy for CRE infection (31). Our results revealed that eravacycline has demonstrated 2- to 4-fold greater potency *in vitro* than tigecycline. The possible reason for this is that the few key distinctions between eravacycline and tigecycline, a fluorine atom at the C-7 position and a pyrrolidinoacetamido group at the C-9 position, take effect. Another reason may be that tigecycline has been used in the clinic for several years, while eravacycline is still under approval in China. Both cefoselis and cefepime belong to the fourth-generation cephalosporins. As expected, the antibacterial activity of cefoselis against *Enterobacterales* was similar to that of cefepime in this study. Our results align with those of the previous studies by other investigators (12, 32).

In summary, this study evaluated the *in vitro* activities of aztreonam-avibactam, eravacycline, and cefoselis against 1,202 *Enterobacterales*, including 119 CRE and 599 MDR isolates, from multiple centers. The results of this large investigation support the clinical development of the three novel antimicrobial agents for the treatment of *Enterobacterales* infections.

## MATERIALS AND METHODS

This study relied on the surveillance program that involved seven subcenters, covering northern China, eastern China, southern China, central China, northeastern China, southwestern China, and northwestern China. The isolates included in the study were randomized and were required to meet the following criteria: the host of the isolates was clinically diagnosed as having a possible infection and only one isolate per patient was included. With reference to the inclusion criterion, each participating center was required to collect nonduplicated *Enterobacterales* isolates from the following specimen sources of inpatients between January 2019 and December 2019: respiratory tract, abdominal cavity, blood, skin and soft tissue, urinary tract, gastrointestinal tract, genital tract, and nervous system. Isolates were sent to the central laboratory. The central laboratory confirmed the species identification of all isolates tested using matrix-assisted laser desorption ionization–time of flight (MALDI-TOF) mass spectrometry.

A total of 1,202 *Enterobacterales* isolates were collected from 26 hospitals in 19 provinces and municipalities that cover seven subcenters, including northern China (*n *= 234), eastern China (*n *= 90), southern China (*n *= 153), central China (*n *= 222), northeastern China (*n *= 147), southwestern China (*n *= 148), and northwestern China (*n *= 208). All antimicrobial susceptibility testing was conducted in a central monitoring laboratory. Aztreonam-avibactam, eravacycline, cefoselis, and 27 other antimicrobial agents were tested using the broth microdilution method (Gram-negative bacteria susceptibility test card; Dier, China) specified by Clinical and Laboratory Standards Institute (CLSI) standards (33). Aztreonam-avibactam and ceftazidime-avibactam were both tested with avibactam at a fixed concentration of 4 μg/mL. The susceptible breakpoints of aztreonam, cefepime, and cefoperazone defined by the CLSI were applied for aztreonam-avibactam, cefoselis, and cefoperazone-sulbactam. The tigecycline and eravacycline susceptibilities were interpreted based on FDA guidelines. The susceptibility rate of colistin was replaced by the nonresistance rate. The susceptibilities of the remaining 24 antimicrobial agents were interpreted according to current CLSI guidelines (34).

MDR *Enterobacterales* isolates were defined as showing acquired resistance to at least one agent in three or more antimicrobial classes according to the combination of CLSI and FDA criteria (tigecycline and eravacycline: FDA criteria, and other antimicrobial agents: CLSI criteria). *Enterobacterales* isolates categorized as CRE were defined as displaying resistance to any carbapenem (imipenem, meropenem, and ertapenem) according to CLSI interpretive criteria. Imipenem was not applied to Proteus spp., *Providencia* spp., or *Morganella* spp. due to their intrinsically elevated MIC values. Subsequently, CRE isolates were tested for the presence of carbapenemase genes (i.e., *bla*_KPC_, *bla*_OXA_, *bla*_NDM_, *bla*_VIM_, *bla*_IMP_, *bla*_GIM_, and *bla*_SPM_) through PCR followed by DNA sequencing. In accordance with CLSI guidelines (34), all *Enterobacterales* isolates were phenotypically screened for possible ESBL production using ceftazidime, ceftriaxone, and aztreonam MICs (any one MIC value of ≥2). Confirmation tests were then conducted for possible ESBL-producing isolates by testing with clavulanate in combination with ceftazidime and cefotaxime. Quality control of ESBL confirmatory test was done using K. pneumoniae ATCC 700603 as a positive control and E. coli ATCC 25922 as a negative control (33). “ESBL screen negative” was defined as meeting at least one of the following criteria: (i) for the isolates, the ceftazidime, ceftriaxone, and aztreonam MIC values were all ≤1 μg/mL, and (ii) the isolates demonstrated a negative result by the ESBL confirmatory test and were nonresistant to ceftazidime, ceftriaxone, and aztreonam. “ESBL screen-positive isolates” were defined as *Enterobacterales* with a positive result for ESBL confirmation tests.

All statistical analyses were performed using Statistical Package for the Social Sciences version 23.0 software (SPSS Inc., Chicago, IL, USA). Pearson’s chi-square (χ^2^) test or correction for continuity was carried out to compare the categorical variables, and a *P* value of ≤0.05 was considered statistically significant. Linear weighted kappa analysis was used for the consistency test for two comparable agents. A weighted kappa coefficient greater than 0.60 indicates strong consistency, a coefficient less than 0.4 indicates poor consistency, and a coefficient of 0.4 to 0.6 indicates moderate consistency.

### Ethics statement.

No human subjects participated in this study. The study was approved by the human research ethics committee of Peking Union Medical College Hospital (PUMCH; no. JS-2581).
