# Supplementary material for: In Vitro Activities of Aztreonam-Avibactam, Eravacycline, Cefoselis, and Other Comparators against Clinical Enterobacterales Isolates: a Multicenter Study in China, 2019
Source: Microbiol Spectr. 2023 May 15;11(3):e04873-22. doi: 10.1128/spectrum.04873-22 (PMC10269566; doi:10.1128/spectrum.04873-22)
Supplement: Supplemental file 1 — Table S1 and Table S2. Download spectrum.04873-22-s0001.pdf, PDF file, 0.1 MB [file spectrum.04873-22-s0001.pdf]

Table S1. In vitro activity of 30 antimicrobial agents against different species and genera of Enterobacterales

Antimicrobial agent	*Escherichia spp.* (*n* = 284)			*Klebsiella spp.* (*n* = 243)			*Citrobacter spp.* (*n* = 82)			*Enterobacter spp.* (*n* = 140)			*Morganella spp.* (*n* = 77)			*Proteus spp.* (*n* = 104)			*Salmonella spp.* (*n* = 169)			*Serratia spp.* (*n* = 97)		
	IC50 (μg/mL)	IC90 (μg/mL)	S[b] (%)	IC50 (μg/mL)	IC90 (μg/mL)	S (%)	IC50 (μg/mL)	IC90 (μg/mL)	S (%)	IC50 (μg/mL)	IC90 (μg/mL)	S (%)	IC50 (μg/mL)	IC90 (μg/mL)	S (%)	IC50 (μg/mL)	IC90 (μg/mL)	S (%)	IC50 (μg/mL)	IC90 (μg/mL)	S (%)	IC50 (μg/mL)	IC90 (μg/mL)	S (%)
AMP[a]	>16	>16	14.79	>16	>16	2.47	>16	>16	4.88	>16	>16	5.71	>16	>16	5.71	>16	>16	36.54	>16	>16	26.63	>16	>16	13.40
CZO	>16	>16	35.92	2	>16	51.03	>16	>16	28.05	>16	>16	4.29	>16	>16	1.30	>16	>16	0.96	4	16	39.05	>16	>16	0.00
CXM	>64	>64	30.99	8	>64	54.32	8	>64	62.20	8	>64	50.00	64	>64	5.19	4	>64	54.81	8	>64	79.88	64	>64	4.12
CAZ	4	64	65.85	≤1	>64	69.96	≤1	>64	69.51	≤1	>64	66.43	16	>64	38.96	1	2	93.27	≤1	16	89.35	≤1	4	90.72
CRO	>64	>64	34.51	≤0.25	>64	58.85	≤0.25	>64	65.85	≤0.25	>64	59.29	1	8	70.13	≤0.25	>64	66.35	≤0.25	>64	86.98	≤0.25	>64	84.54
MOX	≤2	8	92.96	≤2	>32	76.95	≤2	32	80.49	≤2	32	80.00	8	8	93.51	≤2	≤2	96.15	≤2	≤2	98.22	≤2	16	88.66
FEP	4	>16	42.25	≤0.5	>16	62.55	≤0.5	8	85.37	≤0.5	32	75.00	≤0.5	4	87.01	≤0.5	16	73.08	≤0.5	16	87.57	≤0.5	4	89.69
CSE	8	>16	40.14	≤2	>16	60.91	≤2	16	75.61	≤2	>16	66.43	8	>16	42.86	≤2	>16	65.38	≤2	>16	86.39	≤2	4	89.69
FOX	8	>16	73.94	8	>16	68.31	>16	>16	32.93	>16	>16	10.71	8	16	85.71	4	>16	84.62	2	8	93.49	8	>16	55.67
ATM	8	>16	48.94	≤1	>16	67.49	≤1	>16	68.29	≤1	>16	65.71	>16	>16	33.77	≤1	2	95.19	≤1	16	88.76	≤1	>16	87.63
AMC	16/8	>16/8	44.01	8/4	>16/8	61.32	>16/8	>16/8	32.93	>16/8	>16/8	6.43	>16/8	>16/8	1.30	8/8	>16/8	66.35	8/8	>16/8	54.44	>16/8	>16/8	20.62
SAM	16/8	>16/8	70.77	8/4	>16/8	55.14	8/4	>16/8	63.41	>16/8	>16/8	48.57	16/8	>16/8	67.53	8/4	>16/8	81.73	16/8	>16/8	56.21	16/8	>16/8	58.76
CSL	8/4	32/16	83.80	≤0.5/0.25	>64/32	74.90	≤0.5/0.25	16/8	90.24	≤0.5/0.25	>64/32	80.00	8/4	64/32	66.23	2/1	8/4	95.19	8/4	32/16	89.94	≤0.5/0.25	16/8	91.75
AZA	≤1/4	≤1/4	99.65	≤1/4	≤1/4	99.18	≤1/4	≤1/4	100.00	≤1/4	≤1/4	97.86	≤1/4	>16/4	84.42	≤1/4	≤1/4	99.04	≤1/4	≤1/4	100.00	≤1/4	≤1/4	97.94
CZA	≤0.5/4	≤0.5/4	95.07	≤0.5/4	4/4	94.24	≤0.5/4	≤0.5/4	93.90	≤0.5/4	4/4	91.43	≤0.5/4	1/4	98.70	≤0.5/4	≤0.5/4	97.12	≤0.5/4	≤0.5/4	99.41	≤0.5/4	1/4	100.00
TZP	4/4	16/4	92.25	4/4	>128/4	76.54	4/4	64	85.37	4/4	>128/4	75.71	1/4	>128/4	75.32	1/4	2	99.04	4/4	8/4	94.67	1/4	8/4	92.78
ETP	≤0.125	0.5	91.55	≤0.125	>8	76.54	≤0.125	0.5	92.68	≤0.125	4	81.43	≤0.125	≤0.125	97.40	≤0.125	≤0.125	97.12	≤0.125	≤0.125	98.22	≤0.125	2	87.63
IMP	≤0.25	0.5	93.66	≤0.25	>32	79.42	≤0.25	1	96.34	≤0.25	2	87.86	2	4	31.17	4	4	24.04	≤0.25	0.5	99.41	1	1	93.81
MEM	0.064	0.125	94.37	0.064	>32	79.01	0.064	0.25	95.12	0.064	0.5	92.14	0.25	1	97.40	0.25	0.25	98.08	0.064	0.125	99.41	0.25	0.25	94.85
COL[c]	2	2	98.59	2	2	97.12	2	2	97.56	2	4	89.29	>16	>16	2.60	32	32	11.54	2	2	93.49	32	32	11.34
AMK	4	4	97.89	≤1	>32	83.95	≤1	4	100.00	≤1	4	100.00	4	4	100.00	4	16	95.19	2	4	100.00	1	4	100.00

Antimicrobial agent	*Escherichia spp.* (*n* = 284)			*Klebsiella spp.* (*n* = 243)			*Citrobacter spp.* (*n* = 82)			*Enterobacter spp.* (n = 140)			*Morganella spp.* (*n* = 77)			*Proteus spp.* (*n* = 104)			*Salmonella spp.* (*n* = 169)			*Serratia spp.* (*n* = 97)		
	IC50 (μg/mL)	IC90 (μg/mL)	S (%)	IC50 (μg/mL)	IC90 (μg/mL)	S (%)	IC50 (μg/mL)	IC90 (μg/mL)	S (%)	IC50 (μg/mL)	IC90 (μg/mL)	S (%)	IC50 (μg/mL)	IC90 (μg/mL)	S (%)	IC50 (μg/mL)	IC90 (μg/mL)	S (%)	IC50 (μg/mL)	IC90 (μg/mL)	S (%)	IC50 (μg/mL)	IC90 (μg/mL)	S (%)
GEN	2	>8	65.14	≤0.5	>8	72.43	≤0.5	2	93.90	≤0.5	>8	85.00	2	>8	61.04	2	>8	60.58	1	>8	88.17	≤0.5	2	94.85
MNO	4	8	82.75	4	>8	74.07	4	4	90.24	4	>8	77.86	8	>8	46.75	>8	>8	22.12	4	>8	56.80	4	4	93.81
TGC	0.25	0.5	100.00	0.5	1	90.53	0.125	0.5	100.00	0.25	1	98.57	1	4	58.44	4	>4	33.65	0.5	1	98.82	0.25	0.5	97.94
ERV	0.125	0.25	99.65	0.5	1	84.77	0.125	0.125	98.78	0.125	0.5	97.86	1	1	41.56	1	8	33.65	0.5	0.5	93.49	0.125	0.5	95.88
CIP	>2	>2	22.89	0.125	>2	53.50	0.125	2	65.85	≤0.064	>2	65.71	1	>2	44.16	1	>2	41.35	0.25	1	17.16	0.125	1	82.47
LVX	>4	>4	30.63	0.25	>4	57.20	0.25	4	65.85	0.125	>4	69.29	1	>4	44.16	1	8	44.23	0.5	1	17.16	0.25	1	88.66
CHL	8	>16	64.44	8	>16	62.55	8	>16	87.80	8	>16	70.71	16	>16	49.35	16	>16	30.77	8	>16	71.01	8	>16	56.70
SXT	>4/76	>4/76	39.44	≤0.5/9.5	>4/76	63.37	≤0.5/9.5	>4/76	84.15	≤0.5/9.5	>4/76	71.43	≤0.5/9.5	>4/76	62.34	>4/76	>4/76	46.15	≤0.5/9.5	>4/76	78.11	≤0.5/9.5	≤0.5/9.5	94.85
FOS	≤2	8	32.86	8	128	83.13	≤2	8	93.90	8	128	87.14	128	>128	41.56	≤2	>128	65.38	≤2	2	95.27	8	64	96.91

[a]AMP, ampicillin; CZO, cefazolin; CXM, cefuroxime; CAZ, ceftazidime; CRO, ceftriaxone; MOX, moxalactam; FEP, cefepime; CSE, cefoselis; FOX, cefoxitin; ATM, aztreonam; AMC, amoxicillin/clavulanate; SAM, ampicillin/sulbactam; CSL, cefoperazone/sulbactam; AZA, aztreonam/avibactam; CZA, ceftazidime/avibactam; TZP, piperacillin/tazobactam; ETP, ertapenem; IMP, imipenem; MEM, meropenem; COL, colistin; AMK, amikacin; GEN, gentamicin; MNO, minocycline; TGC, tigecycline; ERV, eravacycline; CIP, ciprofloxacin; LVX, levofloxacin; CHL, chloramphenicol; SXT, trimethoprim/sulfamethoxazole; FOS, fosfomycin.

[b]S% means susceptibility rate of strains to corresponding antimicrobial agents.

[c]The susceptibility rate of colistin was replaced by the non-resistance rate

Table S2. MDR and CRE rate among corresponding Enterobacterales isolate with different demographic parameters.

Demographic parameter		MDR rate (%)	CRE rate (%)
Age			
	≤14	52.97	12.38
	15-35	34.00	4.67
	36-60	50.70	9.15
	≥61	53.07	11.32
Specimen source			
	Respiratory tract	51.31	11.52
	Abdominal cavity	53.19	15.96
	Blood	39.42	7.88
	Skin soft tissue	46.30	12.04
	Urinary tract	62.15	10.75
	Gastrointestinal tract	49.73	6.95
	Genital tract	42.86	5.04
	Nervous system	60.42	16.67
Department			
	ICU	63.91	19.55
	Non-ICU	48.08	8.70
